# Cross-Cultural Adaptation, Reliability, and Validity of the Polish Version of the Neck Outcome Score

**DOI:** 10.3390/clinpract13060121

**Published:** 2023-10-30

**Authors:** Nicola Dyrek, Łukasz Pulik, Aleksandra Piwowarczyk, Wiktoria Skała, Nina Grabowska-Mycko, Paweł Łęgosz

**Affiliations:** 1 Student Scientific Association of Reconstructive and Oncology Orthopedics, Department of Orthopedics and Traumatology, Medical University of Warsaw, 02-005 Warsaw, Poland; 2 Department of Orthopedics and Traumatology, Medical University of Warsaw, Lindley 4 Str., 02-005 Warsaw, Polandpawel.legosz@wum.edu.pl

**Keywords:** cervical pain, patient-reported outcome measure, validation study, Poland

## Abstract

This study aimed to translate and psychometrically validate the Neck Outcome Score (NOOS) in the Polish population according to the recommendations of the American Academy of Orthopedic Surgeons Participants completed online version of the NOOS, Neck Disability Index (NDI), and Visual Analogue Scale (VAS) for pain assessment (23 November 2021–9 April 2022). The questionnaires were completed by 57 women and 32 men with cervical spine ailments. A retest was performed after 48 h. The analysis confirmed the high internal consistency (Cronbach’s alpha of 0.95) of the Polish NOOS. No floor/ceiling effects were observed. The Polish NOOS showed a significant Spearman’s coefficient correlation with NDI (0.87; *p* < 0.001) and VAS (0.79; *p* < 0.001). The intraclass correlation coefficient (ICC) for the test–retest was found to be high (0.97). The Polish NOOS can be used for clinical and research purposes as an equivalent to the original version.

## 1. Introduction

It is estimated that the overall prevalence of neck pain is 23.1% of the population, with the highest prevalence in developed countries and urban areas, especially in women [1]. It can be somatic, neuropathic, or a combination of both [2]. A common cause of NP is poor ergonomics at work [3], especially maintaining an unfavorable posture with the cervical spine bent [1]. NP also has significant economic aspects such as pain-related absences from work and the need for medical care [4]. Therefore, it is important to develop tools to help clinicians to assess these conditions. This will allow a proper and reliable evaluation of NP before the start of treatment and adaptation of therapy to the individual needs of the patient. At the end of therapy, questionnaires are used to assess the effectiveness of the treatment and the health progress [2]. For this purpose, PROMs (Patient-Reported Outcome Measures) can be very useful tools, being questionnaires that help to assess health status based on patient self-assessment [5]. Their use can contribute to a better understanding of a patient’s quality of life than traditional OROs (Observer-Reported Outcomes) filled out by a doctor/physiotherapist [6]. Due to the diversity of patients, it is crucial to translate and adapt PROMs into different languages or dialects to ensure appropriate care regardless of the nationality or ethnicity of the patient [2,5,6].

The Neck Outcome Score (NOOS) is a PROM which is used to assess cervical spine dysfunction and pain in scientific research and clinical practice [7]. It is self-administered and can be used in paper or electronic version on a computer/tablet/smartphone. The scale contains 34 items and takes 10–15 min to complete. It is divided into subdomains: ‘Mobility’; ‘Stiffness’; ‘Symptoms’; ‘Sleep disorders’; ‘Daily Activities and Pain’; ‘Participation in everyday life’; and ‘Quality of life’. The patient assigns a score from 0 to 5 for each question, with 0 being no problems and 5 being the highest level of difficulty or pain [7]. The total of the points is converted into a percentage score that indicates the degree of dysfunction [2]. It has already been translated into Arabic, Turkish, and Danish [8,9,10]. The availability of the NOOS in different languages allows researchers to compare results and conduct multicenter international studies or metanalyses among patients with NP [5].

In this article, we present the translation and validation of the Neck Outcome Score (NOOS) questionnaire into Polish [7]. We tested the hypothesis that it can be used for clinical and research purposes as an equivalent to the original English version. The Polish version of the questionnaire was validated in accordance with the American Academy of Orthopedic Surgeons (AAOS) Institute for Work & Health Recommendations for the Cross-Cultural Adaptation of Health Status Measures [11]. The original version of the NOOS was used with the written permission of the authors from the Institute of Sports Science and Clinical Biomechanics, The University of Southern Denmark, Odense [7].

## 2. Materials and Methods

### 2.1. Study Participants

This prospective observational study received a positive opinion from the Bioethics Committee of the Medical University of Warsaw (AKBE/158/2021). The inclusion criteria for selecting patients were as follows: consent to participate in the study, ability to complete the questionnaire electronically in Polish language, age over 18 years old, and current chronic pain in the cervical spine (persisting for a minimum of 3 months) [12]. Individuals were excluded from the study on the basis of past surgical procedures of the cervical segment of the spine, confirmed congenital defects of the spine, or any malignant neoplasms. Patients from the Outpatient Clinic of Department of Orthopedics and Traumatology, Medical University of Warsaw, were sent an emailed online survey (between 23 November 2021 and 9 April 2022) including the Polish NOOS, the Visual Analogue Scale (VAS) for pain assessment and the Polish version of the Neck Disability Index (NDI). The patients were diagnosed (ICD 10: M54.2) by consultant orthopedic surgeons working in the Outpatient Clinic. All participants were informed of the purpose and process of the study and gave their informed consent to participate in it. All respondents’ rights, including the right to personal data protection, were respected. A total of 89 patients participated in the study. All personal data collected have been anonymized according to the Medical University of Warsaw regulations.

### 2.2. NOOS Translation

The translation process consisted of forward translation, reconciliation, back translation, harmonization, and proofreading. Consensus-based standards for the selection of health status measurement instruments (COSMIN), guidelines, and checklists were used to verify the full translation and validation process [11]. Two independent forward translations from English to Polish (T1 and T2) were received from two native Polish translators. The first translator had an academic background (T1) and the second translator did not (T2). A meeting was then held where both translators (T1 and T2) had the opportunity to agree on one common version. This process of synthesis of the initial translations resulted in a unified forward-translated version (T12). Any further disagreements were resolved during the discussion with authors. Next, two independent back-translations into English (BT1 and BT2) were performed blindly to the original NOOS from the unified forward translation version (T12). Native English translators fluent in the target language were employed for these translations. The first one had an academic background (B1) and the second translator did not (B2). Translators did not receive the original English source items or item definitions. The two back-translated versions (BT1 and BT2) were then compared with the unified forward-translated version (UFT). Then, an agreement was reached on a few minor differences that considered the cultural context and the specificity of the spoken Polish language to propose a preliminary version of Polish NOOS. Five patients with NP tested this version to ensure the purpose and meaning of each question was clear and to develop the final Polish version of NOOS after pretesting (Supplementary file S1). The translations are available from the corresponding author on the written request.

### 2.3. Psychometric Validation of the Polish Version of the NOOS

The analysis consisted of evaluating discriminatory power, internal consistency, and potential floor and ceiling effects and then determining the validity of the construct and the reliability of the test–retest. Analysis was carried out using commonly accepted questionnaires, i.e., the NDI [13] and VAS [14]. Participants were asked to complete all questionnaires using Google Forms. The retest was carried out after a 48 h break.

### 2.4. Questionnaires Used in External Validation

The Neck Disability Index (NDI) is a tool for evaluating problems associated with neck pain, limitations in mobility, and difficulties in daily activities. The scale consists of 10 questions about different areas of functioning and the patient assigns points from 0 to 5, assessing the degree of difficulty. The sum of the points gives an overall score that indicates the degree of disability. Higher scores mean greater difficulty and disabilities [2,7].

The Visual Analogue Scale (VAS) can be used to subjectively assess the intensity of pain or other sensations of the patient. It involves presenting the patient with a line where one end means “no pain” or “no discomfort” and the other end means “greatest possible pain” or “greatest pain”. The patient places a marker on the line, indicating the place that corresponds to the intensity of the sensations. The distance from the beginning of the line to the marker is measured and represents the subjective assessment of the intensity of pain or other sensations, where a higher value means a higher intensity of sensations [14].

### 2.5. Statistical Analysis

The analyses were performed using Statistica version 13, developed by TIBCO Inc. based in Palo Alto, CA 94304, USA (2017). The results were considered statistically significant at a significance level of α = 0.05. The normality of quantitative variables was checked using the Shapiro–Wilk test. Normally, quantitative variables are presented as mean ± standard deviation. Non-normally distributed quantitative variables are presented as the median (Me) with the first and third quartiles (P25–P75). Qualitative variables are shown as absolute values and frequency percentages. Cronbach’s alpha coefficient was used to evaluate internal consistency. A high value of the internal consistency coefficient means a value greater than 0.70. An analysis of the effect of the ceiling and floor was also carried out. The effect of the ceiling and floor was considered significant if it exceeded 15% of the population, indicating that the responses were concentrated on the highest or lowest score. To assess the design validity, reliable NOOS items were analyzed using Spearman’s correlation coefficient due to anomalous data distribution. The intraclass correlation coefficient (ICC) was used to assess reliability. ICC values are limited to −1 to 1, where ICC ≈ 1 means very high reliability. The study group size was estimated based on the previous validation studies [8,9,10].

## 3. Results

### 3.1. Descriptive Analysis

A total of 89 patients were included in the study. Patients were asked to complete the online version of NOOS questionnaire, the NDI questionnaire, and the VAS questionnaire. There were 57 (64%) women and 32 (36%) men in the study group. The average age of the subject in the study group was 36.84 years (women—37.51 years; men—35.65 years). The normality analysis showed that none of the variables had a normal distribution.

### 3.2. Psychometric Validation

#### 3.2.1. External Validity

To verify the external validity of the constructed tool, a series of correlation analyses (the Spearman rho) between the results obtained in the Polish NOOS (first measurement) and the results on the VAS scale was carried out. Positive and strong relationships were found between the VAS scale and the subscales of the Polish NOOS in the test and retest measurements (Table 1). Similarly significant correlations were found between the NDI and the subscales of the Polish NOOS (first measurement) (Table 2). Table 3 represents correlations for the total score of the NOOS in first and second measurements with the VAS and NDI.

#### 3.2.2. Reliability Analysis

In order to verify the reliability of Polish NOOS, an analysis was carried out using Cronbah’s alpha coefficient separately for the results from the first and second measurements. Based on the results presented in Table 4, it was found that the coefficients for a ‘Mobility’ and ‘Participation in everyday life’ domain in the first and second measurements were below alpha = 0.70. Other domains’ coefficients were greater than the acceptable treshold for Cronbah’s alpha (0.70)

#### 3.2.3. Intraclass Correlations (ICC)

In addition, the coefficients of intraclass coherence for the measured scales (test–retest) were calculated. Based on the results presented in Table 5, the coefficients of intraclass coherence were found to exceed 0.70 in all domains, except ‘Mobility’ and ‘Participation in Everyday life’.

#### 3.2.4. Floor and Ceiling Effects

None of the patients received the lowest score (0 points) or the highest score (100 points) on the questionnaire. Therefore, neither the floor nor the ceiling effect was found in the questionnaire.

#### 3.2.5. Reliability of the Test–Retest

To verify the constancy of the measured scales over time, an analysis was performed using the Student’s *t*-test for repeated measures. The average results obtained by the subjects in the first and second surveys were compared. Based on the results presented in Table 6, a statistically significant difference was found on the following subscales: ‘Mobility’, ‘Stiffness’, ‘Symptoms’, ‘Sleep disorders’, ‘Daily activities and pain’, ‘Participation in daily life’, and ‘Quality of life’. This means that the results obtained by the subjects after 48 h changed. This may indicate the instability of the scales mentioned above over time.

## 4. Discussion

In recent years, PROMs that have been developed in English-speaking countries for use in international clinical trials have gradually been translated into Polish [2]. Our study was carried out to translate the original English NOOS questionnaire according to international guidelines into an understandable and equivalent Polish version [11]. Since the original version of the NOOS [7] was first developed and validated in 2015, multiple language versions of the questionnaire have become available [8,9,10], allowing researchers to compare study results and perform metanalyses.

Psychometric properties for clinical and research applications, comparable to other studies, are the proof that the equivalence of the Polish and English versions of the questionnaire had high internal consistency with the original (Cronbach’s alpha at the level of 0.95) and turned out to be comparable to the original version. The corresponding internal consistency is comparable to other versions of the NOOS in different languages: Arabic (Cronbach’s α > 0.9) [8], Turkish (Cronbach’s α of 0.85–0.92) [9], Danish (Cronbach’s α of 0.88–0.95) [10]. The total score and the NOOS individual domains had a very high ICC of 0.7, reflecting excellent reliability. The Polish translation of the NOOS showed excellent psychometric properties.

So far, the only PROM available in Polish to assess the impact of NP and other ailments related to the cervical spine on daily living is the NDI [13]. However, the use of the NDI is questioned due to the validation process, i.e., a broad selection of patients, achievement of data saturation, and lack of patient input [7]. The Polish version of the NOOS scale is a useful tool for assessing pain-related symptoms of the cervical spine, which are important to the patient. This study contributes to the area of patient-reported outcome measures to assess the cervical spine problems available in the Polish language.

The availability of the Polish NOOS questionnaire has significant clinical implications, influencing the assessment and treatment of cervical spine disorders in clinical practice. While there is wide range of tools used in the English language for neck-related problems assessment, the availability of such tools in Polish is still very limited [15]. The Polish NOOS allows for a precise evaluation of cervical spine function, enabling better tailoring of therapy to the individual needs of the patient. However, compared to existing measures such as the NDI, the NOOS may be more time-consuming to complete, potentially impacting patients’ willingness for questionnaire use. The NOOS focuses not only on pain intensity but also on how pain affects the daily activities of the patient. This is crucial when planning therapy focused on restoring normal function. The NOOS is relatively straightforward for both patients and medical professionals, increasing its utility in clinical practice. However, compared to more concise tools, simplicity may lead to a loss of diagnostic details. The availability of the NOOS in various languages allows for international comparability of study results, facilitating meta-analysis. The Polish NOOS proves to be a valuable tool in clinical practice, particularly for its accuracy in assessing cervical spine function.

### 4.1. Strengths of the Study

The strengths of this study include the use of standardized methods in all procedures and the large sample of patients with cervical spine complaints. The NOOS questionnaire has been specially developed for patients with cervical pain, which is of particular benefit to this group of patients. Furthermore, an additional advantage of the study is the rigorous translation of the questionnaire and the initial translation assessment by an independent group of four translators.

### 4.2. Limitations

Participants do not necessarily represent the entire spectrum of NP patients with respect to age or cause of symptoms, as the study was carried out online. The average age of the subject in the study group was 36.84 years and reflects mainly middle-aged population. The study by Rowen et al. indicates that the results of PROMs can be different when validation is carried out over the Internet rather than the traditional method [16]. The reliability analysis (Cronbach’s alpha) results for the ‘Mobility’ and ‘Participation in everyday life’ domains in the first and second measurements are below alpha = 0.70, indicating insufficient reliability. As these domains are important for assessing cervical spine problems, the Polish NOOS can be supplemented by other more objective tools assessing mobility, e.g., OROs [17]. The results of the test–retest reliability analysis indicate a statistically significant difference on several subscales between the first and second surveys after a 48 h break. This suggests potential instability in these scales over time; however this can just reflect the subjective feelings of patients changing over time as assessed with PROMs.

## 5. Conclusions

The NOOS questionnaire showed high test–retest consistency and high internal consistency. Translation of this questionnaire into Polish is important and credible in the context of evaluating the results of patients with cervical spine pain. The presented Polish version of the NOOS questionnaire can be used effectively for both clinical and research purposes, being the equivalent of the original English version. Additional validation studies in more diverse populations would benefit the further refinement of the Polish NOOS questionnaire. Also, investigations into the responsiveness of the questionnaire to changes in clinical status over time could bring benefits in the aspect of investigating test–retest reliability of the Polish version of the NOOS.

## Figures and Tables

**Table 1:** Spearman’s rho correlation coefficients for Polish NOOS and VAS.

Individual Domains of NOOS	VAS (Spearman’s rho)
Mobility	0.54 ***
Stiffness	0.75 ***
Symptoms	0.73 ***
Sleep disorders	0.70 ***
Daily activities and pain	0.74 ***
Participation in everyday life	0.44 ***
Quality of life	0.77 ***

NOOS—Neck Outcome Score; NDI—Neck Disability Index; VAS—Visual Analogue Scale; ***—*p* < 0.001.

**Table 2:** Spearman’s rho correlation coefficients for Polish NOOS and NDI.

Individual Domains of NOOS	NDI (Spearman’s rho)
Mobility	0.48 ***
Stiffness	0.73 ***
Symptoms	0.77 ***
Sleep disorders	0.75 ***
Daily activities and pain	0.81 ***
Participation in everyday life	0.55 ***
Quality of life	0.81 ***

NOOS—Neck Outcome Score; NDI—Neck Disability Index; VAS—Visual Analogue Scale; ***—*p* < 0.001.

**Table 3:** Correlation for total score of Polish NOOS with NDI and VAS.

	NOOS 1st	NOOS 2nd	VAS	NDI
NOOS 1st	NA	0.88 ***	0.79 ***	0.87 ***
NOOS 2nd	0.88 ***	NA	0.84 ***	0.86 ***
VAS	0.79 ***	0.84 ***	NA	0.78 ***
NDI	0.87 ***	0.86 ***	0.78 ***	NA

NOOS—Neck Outcome Score; NDI—Neck Disability Index; VAS—Visual Analogue Scale; NA—not applicable; ***—*p* < 0.001.

**Table 4:** Reliability coefficients of the in subsequent measurements of Polish NOOS.

Polish NOOS	Domain	Cronbah’s Alpha
First Measurement	Mobility	0.53
	Stiffness	0.86
	Symptoms	0.85
	Sleep disorders	0.92
	Daily activities and pain	0.95
	Participation in everyday life	−0.09
	Quality of life	0.93
	NOOS total score (1st)	0.95
Second Measurement	Mobility	−0.17
	Stiffness	0.93
	Symptoms	0.88
	Sleep disorders	0.94
	Daily activities and pain	0.97
	Participation in everyday life	−0.14
	Quality of life	0.94
	NOOS total score (2nd)	0.95

NOOS—Neck Outcome Score.

**Table 5:** Intraclass correlation coefficients for the measured scales (test–retest) after a 48 h break.

Individual Domains	ICC
Mobility	0.43
Stiffness	0.93
Symptoms	0.93
Sleep disorders	0.96
Daily activities and pain	0.97
Participation in everyday life	0.33
Quality of life	0.96
NOOS score	0.97

NOOS—Neck Outcome Score.

**Table 6:** Comparison of successive measurements of measured quantitative variables.

NOOS Domains	1st		2nd				95% CI		
	M	SD	M	SD	t	p	LL	UL	d Cohen
Mobility	2.93	0.41	3.33	0.34	−8.56	<0.001	−0.49	−0.31	0.91
Stiffness	1.85	0.83	1.74	0.82	2.13	0.018	0.01	0.22	0.23
Symptoms	2.33	0.86	2.15	0.84	3.76	<0.001	0.08	0.27	0.40
Sleep disorders	1.83	0.86	1.73	0.87	1.94	0.027	0.00	0.20	0.21
Daily activities and pain	1.79	0.85	1.74	0.87	0.80	0.213	−0.06	0.14	0.09
Participation in everyday life	2.50	0.34	2.50	0.33	−0.06	0.477	−0.07	0.06	0.01
Quality of life	1.49	0.75	1.48	0.80	0.13	0.447	−0.08	0.09	0.01

m—mean value; SD—standard deviation; t—Student’s *t*-test result; p—significance; 95% CI—confidence interval for the difference between means; LL and UL—lower and upper limits of the confidence interval.

## Data Availability

The study data can be obtained from the corresponding author.

## Supplementary Materials

The following supporting information can be downloaded at: https://www.mdpi.com/article/10.3390/clinpract13060121/s1, Supplementary file S1.
