# Supplementary material for: Cross-Cultural Adaptation, Reliability, and Validity of the Polish Version of the Neck Outcome Score
Source: Clin Pract. 2023 Oct 30;13(6):1352–9. doi: 10.3390/clinpract13060121 (PMC10660527; doi:10.3390/clinpract13060121)
Supplement: Supplementary file 1 [file clinpract-13-00121-s001.zip › clinpract-2619230-supplementary.pdf]

Neck Outcome Score (NOOS)

Kwestionariusz dla osób z bólami szyi

Imię ________________________________

Data ________________________________

INSTRUKCJE

Poniższy kwestionariusz zawiera pytania dotyczące problemów zdrowotnych z szyją. Pana/Pani odpowiedzi pozwolą nam monitorować stan Pana/Pani zdrowia w codziennym życiu.

Prosimy odpowiedzieć na każde pytanie zaznaczając odpowiedź, która najlepiej opisuje Pana/ Pani stan zdrowia. Prosimy wybrać tylko jedną odpowiedź. Prosimy odpowiedzieć na WSZYSTKIE pytania. Jeśli pytanie nie dotyczy Pana/Pani albo nie odczuwał Pan/nie odczuwała Pani dolegliwości, prosimy wybrać, która z odpowiedzi mogłaby być najwłaściwsza.

RUCHOMOŚĆ

Podczas odpowiedzi na następujące pytania, prosimy pomyśleć o problemach z szyją, które miał Pan/ miała Pani w minionym tygodniu. Określenie „w pełnym zakresie" oznacza zakres ruchomości sprzed wystąpienia objawów. Określając poziom bólu, prosimy odnosić się do najsilniejszego bólu, jakiego Pan/ Pani doświadczył/a.

R1. Jak często był Pan/ była Pani w stanie w pełnym zakresie i bez problemów obracać głową?

Cały tydzień	Często	Czasami	Rzadko	Nigdy
☐	☐	☐	☐	☐

R2. Jak często był Pan/ była Pani w stanie w pełnym zakresie i bez problemów zgiąć szyję lub odchylić głowę do tyłu?

Cały tydzień	Często	Czasami	Rzadko	Nigdy
☐	☐	☐	☐	☐

R3. Jak często był Pan/ była Pani w stanie bez problemów spojrzeć w dół na swoją klatkę piersiową?

Cały tydzień	Często	Czasami	Rzadko	Nigdy
☐	☐	☐	☐	☐

R4. Jak silny ból szyi odczuwał Pan/ odczuwała Pani w zeszłym tygodniu, kiedy obracał Pan/ obracała Pani głową maksymalnie w jedną stronę?

Wcale	Łagodny	Umiarkowany	Silny	Bardzo silny
☐	☐	☐	☐	☐

R5. Jak silny ból szyi odczuwał Pan/ odczuwała Pani w zeszłym tygodniu, kiedy Pan/Pani odchylał/a głowę maksymalnie do tyłu?

Wcale	Łagodny	Umiarkowany	Silny	Bardzo silny
☐	☐	☐	☐	☐

SZTYWNOŚĆ

Sztywność karku oznacza trudności wykonywania ruchów (zginania i odchylania). W jakim stopniu odczuwał Pan/odczuwała Pani sztywność karku w ubiegłym tygodniu?

S1. Jaka była sztywność karku zaraz po przebudzeniu się rano?

Żadna	Niewielka	Umiarkowana	Duża	Nie do wytrzymania
☐	☐	☐	☐	☐

S2. Jaka była sztywność karku później w ciągu dnia?

Żadna	Niewielka	Umiarkowana	Duża	Nie do wytrzymania
☐	☐	☐	☐	☐

OBJAWY

Podczas odpowiedzi na następujące pytania, prosimy pomyśleć o problemach z szyją, które miał Pan/ miała Pani w minionym tygodniu.

O1. Jak opisałby Pan/ opisałaby Pani ból szyi w momencie największego nasilenia?

Brak bólu	Łagodny	Umiarkowany	Silny	Bardzo silny
☐	☐	☐	☐	☐

O2. Czy bolała Pana/ Panią głowa?

Brak bólu	Rzadko	Czasami	Często	Cały tydzień
☐	☐	☐	☐	☐

O3. Jak opisałby Pan/ opisałaby Pani ból głowy w momencie największego nasilenia?

Brak bólu	Łagodny	Umiarkowany	Silny Bardzo	Silny
☐	☐	☐	☐	☐

O4. Czy odczuwał Pan/ odczuwała Pani zawroty głowy?

Wcale	Rzadko	Czasami	Często	Cały tydzień
☐	☐	☐	☐	☐

O5. Czy miał Pan/ miała Pani problemy z koncentracją?

Wcale	Rzadko	Czasami	Często	Cały tydzień
☐	☐	☐	☐	☐

ZABURZENIA SNU

ZS1. W jakim stopniu problemy z szyją zaburzały Pana/ Pani sen w zeszłym tygodniu, kiedy leżał Pan/ leżała Pani w łóżku?

Wcale	Łagodnie	Umiarkowanie	Znacznie	Bardzo mocno
☐	☐	☐	☐	☐

ZS2. W jakim stopniu odczuwał Pan/ odczuwała Pani ból szyi podczas kładzenia się do łóżka?

W żadnym	W niewielkim	W umiarkowanym	W znacznym	W bardzo znacznym
☐	☐	☐	☐	☐

ZS3. W jakim stopniu odczuwał Pan/ odczuwała Pani ból szyi w nocy (np. ból zakłócający sen)?

Wcale	Łagodnie	Umiarkowanie	Znacznie Bardzo	Znacznie
☐	☐	☐	☐	☐

ZS4. Jak często źle Pan spał/ źle Pani spała z powodu bólu szyi?

Nigdy	Bardzo rzadko	Sporadycznie	Często	Przez cały tydzień
☐	☐	☐	☐	☐

CZYNNOŚCI CODZIENNE I BÓL

W jakim stopniu odczuwał Pan/ odczuwała Pani problemy z szyją, kiedy Pan/Pani.

C1. W jakim stopniu odczuwał Pan/ odczuwała Pani problemy z szyją, kiedy Pan/Pani siedział/a bez ruchu dłużej niż jedną godzinę na przykład czytając, oglądając telewizję lub siedząc przy komputerze?

W żadnym W niewielkim W umiarkowanym W znacznym W bardzo znacznym

C2. W jakim stopniu odczuwał Pan/ odczuwała Pani problemy z szyją, kiedy Pan/Pani stał/a przez ponad 30 minut?

W żadnym W niewielkim W umiarkowanym W znacznym W bardzo znacznym

C3. W jakim stopniu odczuwał Pan/ odczuwała Pani problemy z szyją, kiedy Pan/Pani trzymał/a ręce nad głową na przykład podczas ubierania, mycia się lub czesania włosów?

W żadnym W niewielkim W umiarkowanym W znacznym W bardzo znacznym

C4. W jakim stopniu odczuwał Pan/ odczuwała Pani problemy z szyją, kiedy Pan/Pani podnosił/a lub przenosił/a ciężkie przedmioty, takie jak torby z zakupami?

W żadnym W niewielkim W umiarkowanym W znacznym W bardzo znacznym

C5. W jakim stopniu problemy z szyją przeszkadzały Panu/ Pani w codziennych czynnościach w zeszłym tygodniu, kiedy Pan/Pani siedział/a bez ruchu ponad jedną godzinę?

Wcale Łagodnie Umiarkowanie Znacznie Bardzo Znacznie

C6. W jakim stopniu problemy z szyją przeszkadzały Panu/Pani w codziennych czynnościach w zeszłym tygodniu, kiedy Pan/Pani robił/a zakupy?

Wcale Łagodnie Umiarkowanie Znacznie Bardzo Znacznie

C7. W jakim stopniu problemy z szyją przeszkadzały Panu/Pani w codziennych czynnościach w zeszłym tygodniu, kiedy Pan/Pani wykonywał/a lekkie prace domowe, takie jak gotowanie lub wycieranie kurzu?

Wcale Łagodnie Umiarkowanie Znacznie Bardzo Znacznie

C8. W jakim stopniu problemy z szyją przeszkadzały Panu/Pani w codziennych czynnościach w zeszłym tygodniu, kiedy Pan/Pani wykonywał/a cięższe prace domowe, takie jak mycie podłóg lub odkurzanie?

Wcale Łagodnie Umiarkowanie Znacznie Bardzo Znacznie

UDZIAŁ W ŻYCIU CODZIENNYM

Proszę odpowiedzieć na WSZYSTKIE pytania. Jeżeli pytanie Pana/Pani nie dotyczy lub nie doświadczył Pan/Pani wskazanej dolegliwości w minionym tygodniu, prosimy o zaznaczenie najbardziej prawdopodobnej odpowiedzi.

U1. Do jakiego stopnia problemy z szyją utrudniały Pana/Pani życie w minionym tygodniu, kiedy brał Pan/ brała Pani udział w życiu towarzyskim, np. odwiedzał/a Pan/i rodzinę, przyjaciół lub znajomych?

Wcale	Łagodnie	Umiarkowanie	Znacznie	Bardzo znacznie
☐	☐	☐	☐	☐

U2. Do jakiego stopnia problemy z szyją utrudniały Pana/Pani życie w minionym tygodniu, kiedy w czasie wolnym zajmował/a się Pan/i preferowanymi przez siebie aktywnościami (tj. hobby, aktywny wypoczynek)?

Wcale	Łagodnie	Umiarkowanie	Znacznie	Bardzo znacznie
☐	☐	☐	☐	☐

U3. Do jakiego stopnia problemy z szyją utrudniały Pana/Pani życie w minionym tygodniu, kiedy uprawiał/a Pan/i ulubione zajęcia sportowe, takie jak pływanie, jazda na rowerze, bieganie lub tenis?

Wcale	Łagodnie	Umiarkowanie	Znacznie	Bardzo znacznie
☐	☐	☐	☐	☐

U4. Do jakiego stopnia problemy z szyją utrudniały Pana/Pani życie w minionym tygodniu, kiedy zajmował/a się Pan/i pracą lub nauką w domu lub poza nim?

Wcale	Łagodnie	Umiarkowanie	Znacznie	Bardzo znacznie
☐	☐	☐	☐	☐

U5. Czy jest Pan/i w stanie uczestniczyć w preferowanych przez siebie zajęciach fizycznych tak długo, jakby Pan/i chciał/a?

Zawsze	Często	Sporadycznie	Rzadko	Nigdy
☐	☐	☐	☐	☐

U6. Czy był/a Pan/i w stanie uczestniczyć w preferowanych przez siebie zajęciach fizycznych w sposób, w jaki by Pan/i chciał/a?

Zawsze	Często	Sporadycznie	Rzadko	Nigdy
☐	☐	☐	☐	☐

JAKOŚĆ ŻYCIA

J1. Czy problemy z szyją zmusiły Pana/Panią do zmiany stylu życia?

Wcale ☐ Do pewnego stopnia ☐ Umiarkowanie ☐ W znacznym stopniu ☐ W dużym stopniu ☐

J2. Czy doświadcza Pan/Pani ograniczeń w swoim życiu w wyniku problemów z szyją, takich jak unikanie lub ograniczanie pracy, zajęć rekreacyjnych, hobby lub spotkań towarzyskich?

Wcale ☐ Do pewnego stopnia ☐ Umiarkowanie ☐ W znacznym stopniu ☐ W dużym stopniu ☐

J3. Czy Pana/Pani problemy z szyją wpłynęły na relacje z najbliższymi osobami?

Wcale ☐ Do pewnego stopnia ☐ Umiarkowanie ☐ W znacznym stopniu ☐ W dużym stopniu ☐

J4. Czy problemy z szyją mają na Pana/Panią wpływ emocjonalny, na przykład w postaci odczuwania smutku, frustracji lub złości?

Wcale ☐ Do pewnego stopnia ☐ Umiarkowanie ☐ W znacznym stopniu ☐ W dużym stopniu ☐
